# Evaluation of Hospital-Based Hematuria Diagnosis and Subsequent Cancer Risk Among Adults in Denmark

**DOI:** 10.1001/jamanetworkopen.2018.4909

**Published:** 2018-11-21

**Authors:** Mette Nørgaard, Katalin Veres, Anne Gulbech Ording, Jens Christian Djurhuus, Jørgen Bjerggaard Jensen, Henrik Toft Sørensen

**Affiliations:** 1 Department of Clinical Epidemiology, Institute of Clinical Medicine, Aarhus University Hospital, Aarhus, Denmark; 2 Clinical Institute, Aarhus University, Aarhus, Denmark; 3 Department of Urology, Institute of Clinical Medicine, Aarhus University Hospital, Aarhus, Denmark

## Abstract

**Importance:**

Data on the long-term risk of urologic and nonurologic cancer after hematuria diagnosis are sparse. Such data can improve understanding of hematuria and cancer and can provide insight into the clinical course of patients with hematuria.

**Objective:**

To assess the risk of urologic or nonurologic cancer after a hospital-based diagnosis of hematuria.

**Design, Setting, and Participants:**

This cohort study used population-based, nationwide health care databases covering all hospitals in Denmark. The data set included records of all adults (n = 134 173) with an inpatient, outpatient, or emergency department diagnosis of hematuria. The study was conducted from January 1, 1995, to December 31, 2013. Follow-up ended on December 31, 2013. Data analysis was performed from January 16, 2017, to September 18, 2018.

**Main Outcomes and Measures:**

Cumulative risk of cancer was computed, and observed cancer incidence was compared with incidence expected in the general population, using standardized incidence ratios.

**Results:**

Of the 134 173 patients included, 52 367 (39.0%) were women, 81 806 (61.0%) were men, and the median (interquartile range) age was 59 (44-72) years. Within 3 months after hematuria diagnosis, 2647 patients (1.9%) received an invasive bladder cancer diagnosis, 1077 (0.8%) a noninvasive bladder cancer diagnosis, 569 (0.4%) a kidney cancer diagnosis, and 908 (1.1%) a prostate cancer diagnosis. The 3-month cumulative incidence (or absolute risk) of any cancer diagnosis was 4.81% (95% CI, 4.70%-4.93%), the 1-year risk was 6.65% (95% CI, 6.51%-6.78%), and the 5-year risk was 12.34% (95% CI, 12.15%-12.53%). The cumulative incidence of bladder cancer only increased from 1.20% (95% CI, 1.11%-1.30%) after 1 year to 1.36% (95% CI, 1.26%-1.46%) after 5 years of follow-up in women and from 2.93% (95% CI, 2.82%-3.05%) to 3.31% (95% CI, 3.19%-3.44%) in men. For noninvasive bladder cancer, the standardized incidence ratio in the 1 year to less than 5 years of follow-up was 5.39 (95% CI, 4.58-6.30) in patients without initial cystoscopy and was 0.16 (95% CI, 0.04-0.42) in patients with cystoscopy within 3 months after hospital-based diagnosis of hematuria. For kidney cancer, the standardized incidence ratio in the 1 year to less than 5 years of follow-up was 2.63 (95% CI, 2.15-3.18) in patients without cystoscopy and 1.20 (95% CI, 0.87-1.61) in patients with cystoscopy within 3 months after hospital-based diagnosis of hematuria. After 1 year, the risk of gynecologic and colorectal cancers was as expected or even lower, whereas the risk of hematologic malignant neoplasms remained slightly elevated.

**Conclusions and Relevance:**

Increased risk of bladder and kidney cancers even more than 1 year after hospital-based hematuria diagnosis, as well as the slightly elevated risk of invasive bladder cancer after 5 years, may indicate that it is a marker of greater cancer risk; these findings could inform follow-up recommendations for hematuria.

## Key Points

**Question:**

What is the long-term risk of cancer after a hospital-based diagnosis of hematuria?

**Findings:**

In this cohort study of 134 173 Danish adults with a hospital-based hematuria diagnosis, a 2-fold increase in risk of bladder and kidney cancers was found 1 to 5 years after the first hematuria diagnosis but was confined to patients who did not undergo cystoscopy within 3 months of their initial hematuria diagnosis. After 1 year, patients had similar risks of both gynecologic and colorectal cancers as the background population, and the risk of hematologic malignant neoplasms remained slightly elevated.

**Meaning:**

These findings may indicate that hospital-based hematuria diagnosis is a marker of greater cancer risk and may inform follow-up recommendations for hematuria.

## Teaser

This cohort study examines inpatient, emergency department, and outpatient data to assess the risk of urologic or nonurologic cancer among Danish adults with hematuria first diagnosed at the hospital.

## Introduction

Hematuria is a frequent finding and may be a presenting symptom of an underlying urologic malignant neoplasm.^1^ However, long-term data on cancer risk in selected populations with hematuria are sparse and based on small unrepresentative study populations.^2,3,4^ Several aspects of the association between hematuria and cancer risk remain poorly understood. These aspects include whether hematuria is a marker of long-term urologic cancer risk and whether it is associated with increased risk of nonurologic cancers. Colorectal cancer, gynecologic cancer, and hematologic malignant neoplasms all could be associated with hematuria through bladder involvement or bleeding from tumor-induced coagulation disturbances.^5,6,7,8,9,10,11^

Data are needed to improve understanding of both hematuria and cancer and to provide further insight into the clinical course of patients with hematuria, thereby potentially guiding the follow-up of these patients. Therefore, using data from Danish medical databases, we undertook a large population-based assessment of cancer risk in patients with a hospital-based hematuria diagnosis.

## Methods

This study was approved by the Danish Data Protection Agency. According to Danish legislation, registry-based studies do not require informed consent; thus, none was obtained for this analysis. This study followed the Strengthening the Reporting of Observational Studies in Epidemiology (STROBE) reporting guideline.

### Data Sources and Study Population

We conducted this nationwide cohort study in Denmark (with a population of approximately 5.6 million) between January 1, 1995, and December 31, 2013. The Danish National Health Service provides to all residents free tax-funded medical care, including access to general practitioners, hospitals, and outpatient specialty clinics.^12^ All Danish residents have a civil registration number that allows unambiguous linkage between medical databases and administrative registries across the country.^13^ This study used data from the Danish National Patient Registry (DNPR), which includes all admissions to Danish hospitals since 1977 as well as emergency department and outpatient clinic visits since 1995.^14^ Diagnoses are classified in the DNPR according to the *International Classification of Diseases*, *Eighth Revision* (*ICD-8*) until 1994 and the *International Statistical Classification of Diseases and Related Health Problems, Tenth Revision (ICD-10)* thereafter.^14^

We identified all patients with an inpatient, outpatient, or emergency department diagnosis of hematuria recorded in the DNPR between January 1, 1995, and December 31, 2013 (see *ICD-8* and *ICD-10* [hereafter, *ICD*] codes in the eTable in the Supplement). To restrict our cohort to patients with their first hospital-based diagnosis, we excluded those with a hematuria diagnosis before January 1, 1995. We used *ICD-10* codes to categorize the type of hematuria into macroscopic or microscopic hematuria and then examined these types in combination and separately. In addition, we identified patients who underwent cystoscopy and transurethral bladder resection within 3 months before or after their first recorded hematuria diagnosis (see surgical procedures codes in the eTable in the Supplement). Data analysis was performed from January 16, 2017, to September 18, 2018.

### Cancer

We obtained information on incident cancer that occurred before or on December 31, 2013, from the Danish Cancer Registry, which includes all incident cancers in Denmark since 1943.^15^ We excluded patients with any cancer diagnosis (including noninvasive bladder cancer) before the hematuria diagnosis date. We categorized cancers as any cancer and urologic cancers (invasive bladder, noninvasive bladder, kidney, renal pelvic, ureteral, and prostate). Because other cancers could be associated with hematuria by bladder involvement, we included information on gynecologic cancers (uterine, cervical, and ovarian), abdominal cancers (colon, rectal, and liver), and hematologic cancers (leukemia, non-Hodgkin lymphoma, and multiple myeloma) (see *ICD* codes in the eTable in the Supplement). Only the patients’ first cancer diagnosis after hematuria was included. From the cancer registry we also obtained the national rates of incident cancer.

### Covariates

The Danish civil registration number encodes age and sex. For each patient, we identified all urogenital diagnoses recorded in the DNPR prior to the hematuria diagnosis (see *ICD* codes in the eTable in the Supplement). In addition, we identified all diagnoses before the hematuria diagnosis recorded in the DNPR as well as patients with chronic obstructive lung disease, myocardial infarction, connective tissue disease, or moderate or severe kidney disease diagnosis before the hematuria diagnosis. We defined the presence of comorbidity as a record of at least 1 of the chronic diseases included in the Charlson Comorbidity Index^16^ (see *ICD* codes in the eTable in the Supplement).

### Statistical Analysis

We followed up with patients from the date of initial hematuria diagnosis until date of the first cancer diagnosis, death, emigration, or December 31, 2013, whichever came first. We computed median follow-up with interquartile range (IQR). We calculated standardized incidence ratios (SIRs), as a measure of relative risk, to compare observed cancer incidence with expected estimated incidence on the basis of national cancer incidence rates by sex, age (1-year groups), and calendar year (1-year groups). Confidence intervals for SIRs were derived using Byar approximation, assuming that the observed number of cases in a specific category followed a Poisson distribution. We used exact 95% CIs when the observed number of cancers was fewer than 10.^17^ Follow-up periods were 0 to less than 3 months, 3 months to less than 1 year, 1 year to less than 5 years, and 5 or more years. We stratified analyses according to sex, age categories (<30, 30-49, 50-64, 65-79, and ≥80 years), type of hospital contact (inpatient, outpatient, or emergency department), previous urogenital diagnosis (yes or no), and presence of comorbidity (yes or no). We calculated the cumulative incidence proportion (absolute risk) of cancer after 3 months, 1 year, and 5 years of follow-up, treating death as a competing risk.^18^ For the cumulative incidence proportions, we used a log(-log)-transform with a slightly modified version of the SAS macro %CumIncid, to compute SDs and 95% CIs.^19^

Because hematuria can also be a consequence of cystoscopy, we conducted a sensitivity analysis excluding all patients who underwent cystoscopy or transurethral bladder resection within 3 months before their hematuria diagnosis. All statistical analyses were conducted using SAS, version 9.4 (SAS Institute).

## Results

We identified 134 173 patients with a first hospital-based diagnosis of hematuria. This cohort comprised 52 367 women (39.0%) and 81 806 men (61.0%), with a median (IQR) age of 59 (44-72) years. Of these patients, 8834 (6.6%) had macroscopic hematuria and 125 339 (93.4%) had microscopic or unspecified hematuria; 26 696 (19.9%) had a previous urogenital disease (Table 1).

**Table 1.** Observed and Expected Invasive Bladder Cancer Risks and Standardized Incidence Ratios in Patients With a First Hospital-Based Hematuria Diagnosis

Characteristic	Patients, No. (%)	0- to <3-mo Follow-up		3-mo to <1-y Follow-up		1- to <5-y Follow-up		Cancers O/E, No.	≥5-y Follow-up
		Cancers O/E, No.	SIR (95% CI)	Cancers O/E, No.	SIR (95% CI)	Cancers O/E, No.	SIR (95% CI)		SIR (95% CI)
Overall	134 173 (100)	2647/14	186.43 (179.40-193.67)	358/39	9.28 (8.34-10.29)	319/151	2.11 (1.88-2.35)	183/157	1.17 (1.01-1.35)
Women	52 367 (39.0)	568/2	369.66 (339.88-401.35)	58/4	13.43 (10.20-17.36)	65/18	3.59 (2.77-4.57)	29/23	1.24 (0.83-1.79)
Men	81 806 (61.0)	2079/13	164.20 (157.21-171.41)	300/34	8.75 (7.79-9.80)	254/133	1.90 (1.68-2.15)	154/133	1.16 (0.98-1.35)
Age group, y									
0-29	15 102 (11.3)	2/0	2381.2 (288.12-8596.00)	0/0	NA	0/0	NA	0/0	NA
30-49	30 354 (22.6)	73/<1	618.33 (484.65-777.47)	6/<1	16.01 (5.88-34.90)	12/2	5.12 (2.64-8.95)	9/8	1.06 (0.49-2.01)
50-64	37 286 (27.8)	573/2	339.02 (311.82-367.95)	69/5	13.42 (10.44-16.99)	69/28	2.51 (1.95-3.17)	62/53	1.16 (0.89-1.49)
65-79	36 402 (27.1)	1363/8	181.18 (171.68-191.06)	173/21	8.23 (7.05-9.56)	159/88	1.82 (1.54-2.12)	92/83	1.10 (0.89-1.35)
≥80	15 029 (11.2)	636/5	130.70 (120.74-141.27)	110/12	9.12 (7.49-10.99)	79/34	2.32 (1.84-2.89)	20/11	1.78 (1.09-2.75)
No previous urogenital disease	107 477 (80.1)	2279/10	222.14 (213.11-231.45)	274/28	9.76 (8.64-10.99)	272/113	2.40 (2.12-2.70)	141/123	1.15 (0.97-1.35)
Previous urogenital disease	26 696 (19.9)	368/4	93.43 (84.13-103.48)	84/11	7.98 (6.36-9.88)	14/6	1.23 (0.91-1.64)	42/34	1.24 (0.89-1.68)
No comorbidity	93 078 (69.4)	1537/7	214.36 (203.78-225.35)	189/20	9.33 (8.05-10.76)	191/89	2.14 (1.85-2.47)	128/113	1.13 (0.95-1.35)
Comorbidity present	41 095 (30.6)	1110/7	157.94 (148.78-167.51)	169/18	9.22 (7.88-10.72)	128/62	2.05 (1.71-2.44)	55/44	1.26 (0.95-1.63)
Chronic obstructive lung disease present	9616 (7.2)	275/2	193.57 (171.36-217.85)	35/4	9.69 (6.75-13.48)	19/11	1.68 (1.01-2.62)	7/7	1.01 (0.40-2.07)
No chronic obstructive lung disease	124 557 (92.8)	2372/13	185.64 (178.24-193.26)	323/35	9.23 (8.25-10.30)	300/140	2.14 (1.90-2.40)	176/150	1.18 (1.01-1.36)
Previous myocardial infarction	7478 (5.6)	259/2	153.14 (135.06-172.97)	44/4	10.06 (7.31-13.50)	33/15	2.15 (1.48-3.02)	15/11	1.41 (0.79-2.32)
No previous myocardial infarction	126 695 (94.4)	2388/13	190.93 (183.35-198.75)	314/34	9.18 (8.19-10.25)	286/136	2.10 (1.86-2.36)	168/146	1.15 (0.98-1.34)
Connective tissue disease present	3982 (3.0)	66/<1	138.21 (106.89-175.84)	11/1	8.68 (4.33-15.53)	10/5	2.21 (1.06-4.06)	4/4	1.06 (0.29-2.73)
No connective tissue disease	130 191 (97.0)	2581/14	188.11 (180.92-195.51)	347/37	9.30 (8.34-10.33)	309/147	2.10 (1.87-2.35)	179/153	1.17 (1.01-1.36)
Moderate to severe renal disease	4155 (3.1)	86/<1	131.93 (105.52-162.93)	10/2	6.28 (3.01-11.55)	7/5	1.49 (0.60-3.06)	4/3	1.39 (0.38-3.55)
No renal disease	130 018 (96.9)	2561/14	189.06 (181.80-196.52)	348/37	9.41 (8.44-10.45)	312/147	2.13 (1.90-2.37)	178/154	1.16 (1.00-1.35)
Inpatient	35 550 (26.5)	919/5	184.25 (172.53-196.56)	163/13	12.79 (10.90-14.91)	96/45	2.13 (1.73-2.60)	49/40	1.22 (0.90-1.61)
Outpatient	89 036 (66.4)	1474/8	185.55 (176.19-195.27)	157/22	6.98 (5.93-8.16)	196/94	2.09 (1.81-2.40)	120/104	1.16 (0.96-1.38)
Emergency department patient	9587 (7.2)	254/1	200.59 (176.68-226.84)	38/3	11.33 (8.02-15.56)	27/13	2.14 (1.41-3.11)	14/13	1.10 (0.60-1.85)
Microscopic hematuria	125 339 (93.4)	2349/13	175.42 (168.39-182.66)	342/37	9.35 (8.38-10.39)	305/145	2.10 (1.87-2.35)	180/155	1.16 (1.00-1.35)
Macroscopic hematuria	8834 (6.6)	298/1	369.22 (328.49-413.62)	16/2	7.97 (4.55-12.94)	14/6	2.30 (1.23-3.86)	3/2	1.71 (0.35-5.00)
Cystoscopy within 3 mo after or at hematuria diagnosis	66 181 (49.3)	NA	NA	46/17	2.74 (2.01-3.66)	23/64	0.36 (0.23-0.54)	9/58	0.16 (0.07-0.30)
Cystoscopy within 3 mo before hematuria diagnosis	1556 (1.2)	NA	NA	1/<1	1.43 (0.04-7.98)	1/2	0.42 (0.01-2.35)	0/2	NA
No Cystoscopy^a^	66 436 (49.5)	NA	NA	311/21	14.73 (13.14-16.46)	295/85	3.45 (3.07-3.87)	174/97	1.80 (1.54-2.08)

Abbreviations: NA, not applicable; O/E, observed vs expected; SIR, standardized incidence ratio.

^a^ No cystoscopy registered within 3 months before or 3 months after hematuria diagnosis.

### Overall Cancer Risk

Among 134 173 patients, 21 457 had a cancer diagnosis during the median (IQR) follow-up of 5 (2-10) years. The SIR for cancer overall was high during the first 3 months after a hematuria diagnosis (14.15; 95% CI, 13.81-14.50) and then dropped markedly. After more than 5 years of follow-up, an approximately 10% increased cancer risk persisted (SIR, 1.11; 95% CI, 1.09-1.14) (Table 2).

**Table 2.** Observed and Expected Cancer Risks and Standardized Incidence Ratios in 134 173 Patients With a First Hospital-Based Hematuria Diagnosis

Cancer Type	0- to <3-mo Follow-up		3-mo to <1-y Follow-up		1- to <5-y Follow-up		≥5-y Follow-up	
	Cancers O/E, No.	SIR (95% CI)	Cancers O/E, No.	SIR (95% CI)	Cancers O/E, No.	SIR (95% CI)	Cancers O/E, No.	SIR (95% CI)
Any cancer	6425/454	14.15 (13.81-14.50)	2356/1255	1.88 (1.80-1.96)	6072/5096	1.19 (1.16-1.22)	6604/5928	1.11 (1.09-1.14)
Urologic cancers								
Noninvasive bladder	1077/4	242.62 (228.35-257.56)	160/12	13.07 (11.12-15.26)	162/65	2.97 (2.53-3.46)	92/68	1.36 (1.10-1.67)
Kidney	569/7	81.40 (74.85-88.37)	119/19	6.14 (5.09-7.35)	149/78	1.92 (1.63-2.26)	127/87	1.46 (1.22-1.74)
Renal pelvic	196/1	208.51 (180.34-239.84)	63/3	24.35 (18.71-31.15)	67/10	6.49 (5.03-8.25)	23/12	1.96 (1.24-2.95)
Ureteral	61/<1	178.04 (136.18-228.71)	29/1	30.88 (20.68-44.35)	24/4	6.54 (4.19-9.72)	16/4	4.03 (2.30-6.54)
Prostate	908/64	14.18 (13.27-15.13)	371/176	2.11 (1.90-2.34)	826/714	1.16 (1.08-1.24)	880/802	1.10 (1.03-1.17)
Gynecologic cancers								
Uterine	39/5	7.88 (5.61-10.78)	42/14	2.99 (2.16-4.04)	73/59	1.23 (0.97-1.55)	60/76	0.79 (0.60-1.02)
Cervical	23/2	10.19 (6.46-15.29)	0/6	NA	17/26	0.66 (0.38-1.06)	23/28	0.82 (0.52-1.23)
Ovarian	21/4	5.48 (3.39-8.37)	10/11	0.92 (0.44-1.69)	44/45	0.97 (0.71-1.31)	46/54	0.86 (0.63-1.14)
Abdominal cancers								
Colon	122/33	3.73 (3.10-4.46)	92/90	1.03 (0.83-1.26)	353/359	0.98 (0.88-1.09)	448/404	1.11 (1.01-1.22)
Rectal	53/18	3.00 (2.24-3.92)	40/49	0.82 (0.59-1.12)	178/195	0.92 (0.79-1.06)	163/214	0.76 (0.65-0.89)
Liver	24/4	5.96 (3.82-8.87)	18/11	1.63 (0.96-2.57)	66/44	1.49 (1.15-1.89)	57/50	1.14 (0.87-1.48)
Hematologic cancers								
Leukemia	31/10	3.22 (2.19-4.58)	39/26	1.48 (1.05-2.02)	141/105	1.34 (1.13-1.58)	143/116	1.24 (1.04-1.46)
Non-Hodgkin lymphoma	60/11	5.58 (4.26-7.18)	48/30	1.61 (1.19-2.14)	133/119	1.11 (0.93-1.32)	154/137	1.12 (0.95-1.32)
Multiple myeloma	42/4	10.02 (7.22-13.54)	25/12	2.17 (1.40-3.20)	62/46	1.34 (1.03-1.72)	68/52	1.30 (1.01-1.65)

Abbreviations: O/E, observed vs expected; SIR, standardized incidence ratio.

Correspondingly, the 3-month cumulative incidence (or absolute risk) of any cancer diagnosis was 4.81% (95% CI, 4.70%-4.93%), the 1-year risk was 6.65% (95% CI, 6.51%-6.78%; women: 4.02% [95% CI, 3.85%-4.19%]; men: 8.32% [95% CI, 8.13%-8.52%]), and the 5-year risk was 12.34% (95% CI, 12.15%-12.53%; women: 8.62% [95% CI, 8.36%-8.88%]; men: 14.69% [95% CI, 14.43%-14.95%]), considering death as a competing risk (Table 3 and Figure).

**Table 3.** Cumulative Incidence of Selected Types of Cancer After First Hospital-Based Hematuria Diagnosis

Cancer Type	3 mo After Diagnosis, % (95% CI)	1 y After Diagnosis, % (95% CI)	5 y After Diagnosis, % (95% CI)
Any cancer	4.81 (4.70-4.93)	6.65 (6.51-6.78)	12.34 (12.15-12.53)
Women	2.76 (2.63-2.91)	4.02 (3.85-4.19)	8.62 (8.36-8.88)
Men	6.12 (5.96-6.29)	8.32 (8.13-8.52)	14.69 (14.43-14.95)
Urologic cancers			
Invasive bladder	1.98 (1.91-2.06)	2.26 (2.18-2.34)	2.55 (2.47-2.64)
Noninvasive bladder	0.81 (0.76-0.86)	0.93 (0.88-0.98)	1.08 (1.02-1.13)
Kidney	0.43 (0.39-0.46)	0.52 (0.48-0.56)	0.66 (0.61-0.70)
Renal pelvic	0.15 (0.13-0.17)	0.20 (0.17-0.22)	0.26 (0.23-0.29)
Ureteral	0.05 (0.04-0.06)	0.07 (0.06-0.08)	0.09 (0.08-0.11)
Prostate (in men)	1.11 (1.04-1.19)	1.59 (1.50-1.67)	2.87 (2.73-2.98)
Gynecologic cancers (in women)			
Uterine	0.08 (0.05-0.10)	0.15 (0.13-0.20)	0.33 (0.28-0.38)
Cervical	0.05 (0.03-0.08)	0.05 (0.03-0.08)	0.08 (0.05-0.13)
Ovarian	0.05 (0.03-0.05)	0.06 (0.05-0.08)	0.15 (0.13-0.20)
Abdominal cancers			
Colon	0.09 (0.08-0.11)	0.16 (0.14-0.19)	0.50 (0.46-0.54)
Rectal	0.04 (0.03-0.05)	0.07 (0.06-0.09)	0.24 (0.21-0.27)
Liver	0.02 (0.01-0.03)	0.03 (0.02-0.04)	0.10 (0.08-0.11)
Hematologic cancers			
Leukemia	0.02 (0.02-0.03)	0.05 (0.04-0.07)	0.19 (0.16-0.21)
Non-Hodgkin lymphoma	0.05 (0.03-0.06)	0.08 (0.07-0.10)	0.21 (0.18-0.23)
Multiple myeloma	0.03 (0.02-0.04)	0.05 (0.04-0.06)	0.11 (0.09-0.13)

**Figure.**
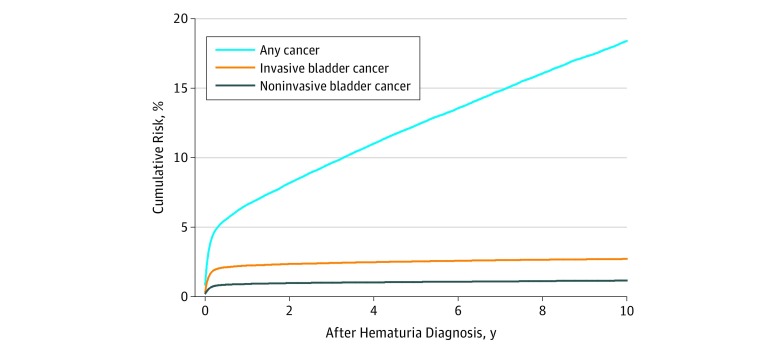
Cumulative Cancer Incidence Cumulative incidence of any cancer, invasive bladder cancer, and noninvasive bladder cancer within 10 years of follow-up among adults with a first hospital-based hematuria diagnosis, with other cancers and death treated as competing risks.

Among the 8834 patients with macroscopic hematuria, 748 (8.5%) had a cancer diagnosis within the first 3 months, compared with 5677 (4.5%) among the 125 339 patients with microscopic hematuria. The 5-year cumulative incidence was 15.76% (95% CI, 14.87%-16.67%) for macroscopic hematuria and was 12.09% (95% CI, 11.90%-12.28%) for microscopic hematuria. Accordingly, patients with macroscopic hematuria had a particularly high SIR of any cancer in the first 3 months (SIR, 26.14; 95% CI, 24.30-28.08) but did not have increased cancer risk more than 5 years after hematuria diagnosis (SIR, 0.87; 95% CI, 0.66-1.12).

### Bladder Cancer

During the first 3 months, 2647 patients (1.9%) received an invasive bladder cancer diagnosis (Table 1). The 3-month risk was 1.09% (95% CI, 1.00%-1.18%) in women and 2.55% (95% CI, 2.45%-2.66%) in men (Table 4). In addition, 1077 patients (0.8%) received a noninvasive bladder cancer diagnosis.

**Table 4.** Cumulative Incidence of Invasive Bladder Cancer After First Hospital-Based Hematuria Diagnosis

Patient Characteristic	3 mo After Diagnosis, % (95% CI)	1 y After Diagnosis, % (95% CI)	5 y After Diagnosis, % (95% CI)
Total	1.98 (1.91-2.06)	2.26 (2.18-2.34)	2.64 (2.55-2.73)
Women	1.09 (1.00-1.18)	1.20 (1.11-1.30)	1.36 (1.26-1.46)
Men	2.55 (2.45-2.66)	2.93 (2.82-3.05)	3.31 (3.19-3.44)
Age group, y			
0-29	0.01 (0.00-0.05)	0.01 (0.00-0.05)	0.01 (0.00-0.05)
30-49	0.24 (0.19-0.30)	0.26 (0.21-0.33)	0.31 (0.25- 0.38)
50-64	1.54 (1.42-1.67)	1.74 (1.61-1.87)	1.96 (1.82-2.11)
65-79	3.76 (3.57-3.96)	4.25 (4.05-4.47)	4.80 (4.58-5.03)
≥80	4.25 (3.93-4.58)	5.00 (4.66-5.36)	5.63 (5.26-6.01)
Inpatient	2.60 (2.43-2.77)	3.07 (2.89-3.25)	3.38 (3.19-3.57)
Outpatient	1.66 (1.58-1.75)	1.85 (1.76-1.94)	2.13 (2.03-2.23)
Emergency department patient	2.66 (2.35-2.99)	3.06 (2.73-3.42)	3.38 (3.03-3.76)
No previous urogenital disease	2.15 (2.06-2.24)	2.42 (2.32-2.51)	2.73 (2.63-2.83)
Previous urogenital disease	1.38 (1.25-1.52)	1.70 (1.56-1.85)	1.94 (1.78-2.10)
No comorbidity	1.66 (1.58-1.74)	1.87 (1.78-1.96)	2.12 (2.03-2.22)
Comorbidity present	2.71 (2.56-2.87)	3.14 (2.98-3.31)	3.53 (3.35-3.72)
Microscopic hematuria	1.88 (1.81-1.96)	2.16 (2.08-2.25)	2.46 (2.37-2.55)
Macroscopic hematuria	3.40 (3.04-3.79)	3.60 (3.22-4.00)	3.89 (3.49-4.33)

The risk of invasive bladder cancer remained 9-fold higher during the 3 months to less than 1 year of follow-up and 2-fold higher during the 1 year to less than 5 years of follow-up (Table 1). After 5 years of follow-up, an approximately 20% increased risk of invasive bladder cancer persisted. At the same time, the cumulative incidence of bladder cancer only increased from 1.20% (95% CI, 1.11%-1.30%) after 1 year to 1.36% (95% CI, 1.26%-1.46%) after 5 years of follow-up in women and from 2.93% (95% CI, 2.82%-3.05%) to 3.31% (95% CI, 3.19%-3.44%) in men (Table 4 and Figure). Among the 15 102 patients (11.3%) younger than 30 years, only 2 cases of invasive bladder cancer were detected in the first 3 months and 0 cases during the remaining follow-up.

Three months after hematuria diagnosis, 298 patients with macroscopic hematuria (cumulative incidence, 3.40%; 95% CI, 3.04%-3.79%) and 2349 patients with microscopic hematuria (cumulative incidence, 1.88%; 95% CI, 1.81%-1.96%) had an invasive bladder cancer diagnosis. From 3 months to 5 years, invasive bladder cancer risk increased to 3.89% (95% CI, 3.49%-4.33%) in patients with macroscopic hematuria and to 2.46% (95% CI, 2.37%-2.55%) in patients with microscopic hematuria.

Within 3 months after hematuria diagnosis (including index admission), 66 181 patients (49.3%) underwent cystoscopy and an additional 1556 patients (1.2%) had a cystoscopy within 3 months before hematuria diagnosis. These patients had a substantially lower risk of invasive bladder cancer compared with patients without a cystoscopy recorded in these periods. The SIR in the 1 year to less than 5 years of follow-up was 0.42 (95% CI, 0.01-2.35) among patients with cystoscopy within 3 months before hematuria, was 0.36 (95% CI, 0.23-0.54) among patients with cystoscopy within 3 months after hematuria, and was 3.45 (95% CI, 3.07-3.87) among those without initial cystoscopy (Table 1). For noninvasive bladder cancer, we observed a similar pattern with an SIR in the 1 year to less than 5 years of follow-up of 5.39 (95% CI, 4.58-6.30) in patients without initial cystoscopy and 0.16 (95% CI, 0.04-0.42) in patients with cystoscopy within 3 months after first hematuria diagnosis.

### Other Urologic Cancers

In total, 569 patients (0.4%) received a kidney cancer diagnosis within 3 months after their hematuria diagnosis (Table 2). The SIR in the 3 months to less than 1 year of follow-up was 6.14 (95% CI, 5.09-7.35) and in the 1 year to less than 5 years of follow-up was 1.92 (95% CI 1.63-2.26). After 5 or more years of follow-up, the risk of kidney cancer remained almost 50% high. Yet, the cumulative incidence of kidney cancer 3 months after hematuria diagnosis was 0.33% (95% CI, 0.28%-0.38%) in women and 0.49% (95% CI, 0.44%-0.54%) in men, whereas the 5-year risks were 0.49% (95% CI, 0.44%-0.54%) in women and 0.78% (95% CI, 0.72%-0.84%) in men. For kidney cancer, the SIR in the 1 year to less than 5 years of follow-up was 2.63 (95% CI, 2.15-3.18) in patients without cystoscopy and 1.20 (95% CI, 0.87-1.61) in patients with cystoscopy within 3 months after first hematuria diagnosis.

After 3 months, renal pelvic cancer was diagnosed in 196 patients (cumulative incidence, 0.15%; 95% CI, 0.13%-0.17%) and ureteral cancer in 61 patients (cumulative incidence, 0.05%; 95% CI, 0.04%-0.06%). After 5 or more years, the risk of these cancers remained increased. The SIR for renal pelvic cancer was 1.96 (95% CI, 1.24-2.95), whereas the SIR for ureteral cancer was 4.03 (95% CI, 2.30-6.54) (Table 2). Still, the 5-year risks of these cancers were low: 0.3% for renal pelvic cancer and less than 0.1% for ureteral cancer. Except in 7 cases, all renal pelvic and ureteral cancers detected during follow-up occurred in patients without a cystoscopy within 3 months after hematuria diagnosis.

Within 3 months of hematuria diagnosis, 908 men (1.1%) received a prostate cancer diagnosis (cumulative incidence, 1.11%; 95% CI, 1.04%-1.19%). During the 1 year to less than 5 years of follow-up after hematuria diagnosis, a 16% increased risk of prostate cancer remained (Table 2). The SIR in the 1 year to less than 5 years of follow-up was only increased in patients without cystoscopy within 3 months of hematuria (SIR, 1.38; 95% CI, 1.27-1.50). Patients who underwent cystoscopy within 3 months of hematuria had an SIR in the 1 year to less than 5 years of follow-up of 0.68 (95% CI, 0.22-1.57).

### Other Cancers

Women were at an increased risk of a gynecologic cancer diagnosis within the first 3 months after hematuria diagnosis. Still, the 3-month cumulative incidence of the specific cancers ranged between only 0.05% and 0.08%. After more than 3 months of follow-up, the risks of cervical and ovarian cancers were lower than expected and remained low even 5 or more years after hematuria diagnosis (cervical SIR, 0.82 [95% CI, 0.52-1.23] and ovarian SIR, 0.86 [95% CI, 0.63-1.14]) (Table 2).

Hematuria was associated with a 4- to 6-fold increased 3-month risk of colorectal or liver cancer (Table 2). After more than 3 months, the incidence of colorectal cancer was similar to or lower than the incidence in the general population.

Hematologic malignant neoplasms also may present with bleeding episodes. We found that risks of leukemia, lymphoma, and multiple myeloma remained slightly increased during the entire follow-up period. Still, the cumulative incidence of these cancers after 5 years of follow-up were only 0.1% to 0.2% (Table 2).

### Sensitivity Analysis

In total, 1556 patients (1.2%) had a cystoscopy and an additional 740 patients (0.6%) had a transurethral bladder resection within 3 months before their hematuria diagnosis. Excluding these patients did not substantially change the overall SIRs for invasive bladder cancer. The SIR in less than 3 months of follow-up was 187.33 (95% CI, 180.20-194.67), in 3 months to less than 1 year of follow-up was 9.45 (95% CI, 8.50-10.49), in 1 year to less than 5 years of follow-up was 2.13 (95% CI, 1.90-2.38), and in 5 or more years of follow-up was 1.18 (95% CI, 1.02-1.37). For any cancer, the SIR in the 1 year to less than 5 years of follow-up was 1.20 (95% CI, 1.17-1.23). For other urologic cancers and for gynecologic, abdominal, or hematologic cancers, the SIRs did not change substantially either.

## Discussion

As expected, we found an increased risk of cancer, particularly bladder and other urologic cancers, within the first 3 months after diagnosis of hematuria. After more than 5 years of follow-up, the risk of both bladder and other urologic cancers remained slightly elevated. However, the increase in cumulative incidence during 1 to 5 years of follow-up was modest, and the increased risk of urologic cancers was confined to patients without documented cystoscopy. Although the risk of gynecologic and colorectal cancers was increased in the first 3 months after hematuria diagnosis, it was as expected or even lower 1 year after hematuria diagnosis. For liver cancer and hematologic malignant neoplasms, a slightly elevated risk persisted throughout follow-up, but the cumulative incidence was small.

In Denmark, patients with a hospital-based (inpatient or outpatient) diagnosis of hematuria usually have been previously evaluated and referred by a general practitioner for a hospital consultation. As the general practitioner may have ruled out common benign sources of hematuria, referred patients are likely to have a greater risk of underlying cancer compared with patients seen only in primary care. This may explain why the 1.9% 1-year risk of invasive bladder cancer found in this study was higher than the 0.7% 3-year risk of urologic cancer found among patients with microscopic hematuria in the 2004 to 2005 study of the Kaiser Permanente Southern California Health Plan.^20^

However, the risk of bladder cancer in our study was much lower than the 12% 6-month risk reported in the United Kingdom both by Khadra et al^21^ among 1930 patients referred to a hematuria clinic from 1994 to 1997 and by Edwards et al^22^ among 4020 patients referred by general practitioners to another hematuria clinic from 1998 to 2003. Similarly, a previous Danish study of 1577 patients with hematuria referred from general practice to a single hospital reported a cancer detection rate of 14.5%.^23^ Danish coding practices may explain this difference, given that patients with an obvious underlying condition associated with hematuria could receive a code for the condition without receiving a hematuria diagnosis code. Therefore, the present hematuria population likely primarily consisted of patients whose initial examination ruled out an obvious source for hematuria. Patients in whom the initial cystoscopy did not detect any bladder tumors could also be more likely to get the hematuria diagnosis than patients in whom a bladder tumor was detected that led to a cancer diagnosis instead. This situation could at least partly explain the lower long-term risk of bladder cancer in patients who underwent an initial cystoscopy, because patients without bladder tumors are likely to have a lower long-term risk of bladder cancer than patients with an unknown bladder status. Another explanation could be that initial cystoscopy may lead to the detection of both invasive and noninvasive bladder cancer. Prompt detection of noninvasive bladder tumors offers the potential for treatment and prevention of invasion, which may be associated with reduced long-term risk of invasive bladder cancer.

The American Urological Association guideline for asymptomatic microscopic hematuria recommends yearly evaluation for urinary tract cancer in case of persistent hematuria after a negative urologic workup.^24^ We identified few additional cases of invasive bladder cancer more than 1 year after hematuria diagnosis among patients who underwent a cystoscopy within 3 months before or after their hematuria diagnosis. Because the American Urological Association recommends cystoscopy for all patients older than 35 years with hematuria,^24^ follow-up for more than 1 year may not be needed. This recommendation accords with suggestions by Mishriki et al^3^ that patients with monosymptomatic hematuria who have undergone a thorough initial investigation with normal results do not need further follow-up.

Inclusion of nonurologic cancers in this study adds to the existing literature. The 12-fold increased risk of any cancer during the first 3 months after the initial hematuria diagnosis likely indicates that hematuria can be the presenting symptom of an occult cancer or that an occult cancer can be an incidental finding during diagnostic workup for hematuria. For rectal and gynecologic cancers, the initial increased risk of cancer was succeeded by a compensatory decreased risk, which was sustained during more than 5 years of follow-up.

### Limitations

This study has limitations. We restricted the study to patients with a hospital-based hematuria diagnosis, which may have produced some bias by referral. However, patients with hematuria with no hospital contact should not be at greater risk of developing urologic cancer than those included in this study. The accuracy of hematuria diagnosis codes in the DNPR is another concern.^14^ As mentioned, hematuria codes are likely predominantly used if the initial examination ruled out an obvious underlying condition for the hematuria; therefore, we do not expect the codes to capture all patients referred to the hospital if, for example, the underlying cancer is coded instead. This would make us underestimate the 3-month risk of cancer, and long-term risk would be less affected.

However, the positive predictive value of coding bleeding disorders among women undergoing a gynecologic surgical procedure has been estimated to be 94%,^25^ and we expect the positive predictive value of hematuria to be similarly elevated. We therefore do not think that misclassification of hematuria represented a major source of bias in this study.

Another study weakness is the lack of information on whether hematuria was accompanied by other symptoms, given that hematuria without other symptoms is associated with a substantially lower cancer risk.^23,26^ In addition, we lacked information on the severity of microscopic hematuria and whether hematuria was identified by the dipstick test only. Therefore, we could not assess whether cancer risk differed by severity of microscopic hematuria. Consistent with previous studies,^22,27,28^ this study found that patients with macroscopic hematuria had a higher 3-month risk of bladder cancer compared with patients with microscopic hematuria. We did not have information on smoking, which is a well-known risk factor for cancer, including bladder cancer.^29^ We did not, however, detect any major difference in invasive bladder cancer risks between patients with hematuria with chronic obstructive lung disease (which may be a crude marker of smoking) and those without.

## Conclusions

A hospital-based hematuria diagnosis may be a marker of an increased risk of cancer. Three months to more than 5 years after the first hematuria diagnosis, the risk of urologic cancers and hematologic malignant neoplasms remained slightly elevated. Nevertheless, the cumulative incidence of cancer was low and the increased risk of urologic cancers was confined to patients without documented cystoscopy within 3 months before or after hematuria diagnosis.

## Notes

Supplement. **eTable.** Diagnosis and Procedure Codes Used in the Study
