# Supplementary material for: Evaluation of Hospital-Based Hematuria Diagnosis and Subsequent Cancer Risk Among Adults in Denmark
Source: JAMA Netw Open. 2018 Nov 21;1(7):e184909. doi: 10.1001/jamanetworkopen.2018.4909 (PMC6324388; doi:10.1001/jamanetworkopen.2018.4909)
Supplement: Supplement. — eTable. Diagnosis and Procedure Codes Used in the Study [file jamanetwopen-1-e184909-s001.pdf]

Supplementary Online Content

Nørgaard M, Veres K, Ording AG, Djurhuus JC, Jensen JB, Sørensen HT. Evaluation of hospital-based hematuria diagnosis and subsequent cancer risk among adults in Denmark. *JAMA Netw Open.* 2018;1(7): e184909. doi:10.1001/jamanetworkopen.2018.4909

eTable. Diagnosis and Procedure Codes Used in the Study

This supplementary material has been provided by the authors to give readers additional information about their work.

©2018 Nørgaard M et al. *JAMA Network Open*

eTable. Diagnosis and Procedure Codes Used in the Study

Disease	ICD-8 codes	ICD-10 codes
Hematuria	789.3, 789.4	R31.9 (unspecified), R319A (macroscopic), R319B (microscopic)
Any tumor	140-194	C00-C75, D303
Invasive bladder cancer		C67.9
Non-invasive bladder cancer*		D30.3
Kidney cancer		C64
Renal pelvis cancer		C65
Ureteral cancer		C66
Prostate cancer		C61
Uterine cancer		C54–C55
Cervical cancer		C53
Ovarian cancer		C56, C570–574
Colon cancer		C18–C19
Rectal cancer		C20
Liver cancer		C22
Leukemia		C91–C95
Non–Hodgkin lymphoma		C82–86, C88 (excl. C826, C840, C841, C848, C863, C866, C884B)
Multiple myeloma		C90
Previous urogenital disease	403, 404, 580-583, 584, 590.09, 593.19, 753.10-753.19, and 792	I12, I13, N00-42, N70-89
Cystoscopy	Nordic Classification of Surgical Procedures code: KUKC	
Transurethral bladder resection (TUR-B)	Nordic Classification of Surgical Procedures code: KCD32.	
Comorbidities:		
Myocardial infarction	410	I21;I22;I23
Chronic pulmonary disease	490-493; 515-518	J40-J47; J60-J67; J68.4; J70.1; J70.3; J84.1; J92.0; J96.1; J98.2; J98.3
Connective tissue disease	712; 716; 734; 446; 135.99	M05; M06; M08; M09;M30;M31; M32; M33; M34; M35; M36; D86
Moderate to severe renal disease	403; 404; 580-583; 584; 590.09; 593.19; 753.10-753.19; 792	I12; I13; N00-N05; N07; N11; N14; N17-N19; Q61

Other comorbidities included in the Charlson comorbidity Index:		
Congestive heart failure	427.09; 427.10; 427.11; 427.19;<br>428.99; 782.49	I50; I11.0; I13.0; I13.2
Peripheral vascular disease	440; 441; 442; 443; 444; 445	I70; I71; I72; I73; I74; I77
Cerebrovascular disease	430-438	I60-I69; G45; G46
Dementia	290.09-290.19; 293.09	F00-F03; F05.1; G30
Ulcer disease	530.91; 530.98; 531-534	K22.1; K25-K28
Mild liver disease	571; 573.01; 573.04	B18; K70.0-K70.3; K70.9; K71; K73; K74; K76.0
Diabetes type1<br><br>Diabetes type2	249.00; 249.06; 249.07; 249.09<br>250.00; 250.06; 250.07; 250.09	E10.0, E10.1; E10.9<br><br>E11.0; E11.1; E11.9
Hemiplegia	344	G81; G82
Diabetes with end organ damage	249.01-249.05; 249.08<br>250.01-250.05; 250.08	E10.2-E10.8 E11.2-E11.8
Moderate to severe liver disease	070.00; 070.02; 070.04; 070.06;<br>070.08; 573.00; 456.00-456.09	B15.0; B16.0; B16.2; B19.0; K70.4; K72; K76.6; I85
AIDS	079.83	B21-B24

*According to Danish coding practice, only invasive bladder tumors are coded as C67.9. Non-invasive bladder cancers are coded as D30.3.

©2018 Nørgaard M et al. *JAMA Network Open*
